# EAU20 Virtual

**DOI:** 10.1007/s13629-020-00305-8

**Published:** 2020-09-02

**Authors:** M.T.W.T. (Tycho) Lock

**Affiliations:** Tijdschrift voor Urologie, Utrecht, Nederland

## Abstract

Sinds de COVID-19-uitbraak lijkt onze wereld heel anders te functioneren. Wie had op nieuwjaarsmorgen 2020 kunnen bedenken hoe anders het nieuwe jaar vanaf februari zou gaan verlopen. Terwijl wij midden in de naweeën van Brabants carnaval en terugkomers van de wintersport zaten, vond president Donald Trump het een ‘klein’ Chinees griepje. In zijn kielzog kwetterde president Jair Bolsenaro in Brazilië eenzelfde liedje. Beiden weten, nu ruim vier maanden verder, de laatste zelf net gerecupereerd van corona, dat de werkelijke feiten fors dramatischer zijn.

Sinds de COVID-19-uitbraak lijkt onze wereld heel anders te functioneren. Wie had op nieuwjaarsmorgen 2020 kunnen bedenken hoe anders het nieuwe jaar vanaf februari zou gaan verlopen. Terwijl wij midden in de naweeën van Brabants carnaval en terugkomers van de wintersport zaten, vond president Donald Trump het een ‘klein’ Chinees griepje. In zijn kielzog kwetterde president Jair Bolsenaro in Brazilië eenzelfde liedje. Beiden weten, nu ruim vier maanden verder, de laatste zelf net gerecupereerd van corona, dat de werkelijke feiten fors dramatischer zijn. Op moment van schrijven van dit editorial (tijdens waarneming in Curaçao) zijn er in de Verenigde Staten bijna 5.000.000 bewezen besmette personen en bijna 160.000 doden te betreuren. Dat staat gelijk met de *gehele* bevolking van Curaçao of steden als Enschede of Arnhem.

Dit nieuwe virus heeft een enorme impact op elk onderdeel van ons maatschappelijk functioneren, dus ook op onze wetenschappelijke nascholing. Zowel de EAU als de AUA zag zich genoodzaakt hun congres te annuleren, hoewel alle wetenschappelijke data reeds waren aangeleverd. Het tekent het professioneel management van beide organisaties dat zij in staat waren alle informatie ‘corona-proof’ via de digitale weg met ons te delen.

drs. M.T.W.T. (Tycho) Lock Utrecht, Nederland m.t.w.t.lock@umcutrecht.nl

Digitaal is natuurlijk corona-safe, maar niet gezellig. Bijpraten met nationale en internationale collega’s is natuurlijk niet aan de orde, laat staan dwalen over een uitgebreide ‘exhibition’ of aangenaam dineren in de avonduren. Juist nu: de jaarlijkse EAU-meeting in Nederland! Dit alles zou nota bene in ons eigen Amsterdam gebeuren…, helaas! De bekende Nederlandse ‘filosoof’ Johan Cruijff had daar een uitdrukking voor: ‘Elk nadeel heb z’n voordeel.’ Dankzij corona is het mogelijk webcasts en andere samenvattingen van EAU en AUA te lezen en te horen wanneer je maar wilt, ook in je luie stoel.

Het *Tijdschrift voor Urologie* had reeds het initiatief genomen tot het samenstellen van een speciale bijdrage naar aanleiding van de EAU in Amsterdam. Ook dit initiatief werd wreed verstoord door de corona-uitbraak. Toen duidelijk werd dat in het derde weekend van juli de EAU zou starten met de digitale versie van de *Amsterdam meeting,* konden wij acht ‘teams’ vinden die in vakantietijd én aanwezig én bereid waren een verslag te maken in nauwelijks twee weken., waarvoor enorme hulde van redactie en uitgever! Helaas moest het team ‘gevorderd prostaatcarcinoom’ door droevige familieomstandigheden op het laatste moment afhaken.

De redactie hoopt van u te vernemen hoe u deze speciale bijlage waardeert (via een mini-enquête), om te kunnen beoordelen of dit een vaste septemberuitgave moet worden. Een groot voordeel is dat u tot in lengte van jaren via https://www.springer.com/journal/13629 gratis toegang hebt tot de tekst, net als tot alle overige uitgaven van ons tijdschrift.

